# Delayed diagnosis, greater disease burden: primary aldosteronism in a Brazilian cohort

**DOI:** 10.1210/jendso/bvag199

**Published:** 2026-08-28

**Authors:** Jessica Lopes Montozo, Paula Conde Lamparelli Elias, Anne Elise Alexandre, Carlos Augusto Fernandes Molina, Valdair Francisco Muglia, Jorge Elias, Margaret Castro, Ayrton C Moreira, Livia M Mermejo

**Affiliations:** Department of Internal Medicine, Ribeirao Preto Medical School—University of Sao Paulo, Ribeirão Preto, SP 14049-900, Brazil; Department of Internal Medicine, Ribeirao Preto Medical School—University of Sao Paulo, Ribeirão Preto, SP 14049-900, Brazil; Department of Internal Medicine, Ribeirao Preto Medical School—University of Sao Paulo, Ribeirão Preto, SP 14049-900, Brazil; Department of Surgery and Anatomy, Ribeirao Preto Medical School—University of Sao Paulo, Ribeirão Preto, SP 14049-900, Brazil; Department of Medical, Imaging, Hematology and Oncology, Ribeirao Preto Medical School—University of Sao Paulo, Ribeirão Preto, SP 14049-900, Brazil; Department of Medical, Imaging, Hematology and Oncology, Ribeirao Preto Medical School—University of Sao Paulo, Ribeirão Preto, SP 14049-900, Brazil; Department of Internal Medicine, Ribeirao Preto Medical School—University of Sao Paulo, Ribeirão Preto, SP 14049-900, Brazil; Department of Internal Medicine, Ribeirao Preto Medical School—University of Sao Paulo, Ribeirão Preto, SP 14049-900, Brazil; Department of Internal Medicine, Ribeirao Preto Medical School—University of Sao Paulo, Ribeirão Preto, SP 14049-900, Brazil

**Keywords:** primary aldosteronism, hypertension, severity classification, delayed diagnosis, lateralized and bilateral primary aldosteronism, adrenalectomy

## Abstract

**Background:**

Primary aldosteronism (PA) is frequently underdiagnosed, resulting in prolonged exposure to aldosterone excess. In Brazilian cohorts, delayed diagnosis has been associated with high prevalence of hypokalemia, suggesting severe disease at presentation. We evaluated PA severity and its association with PA subtype using the Primary Aldosteronism Severity Classification (PASC).

**Methods:**

PA patients were retrospectively evaluated over a 25-year period at a tertiary university hospital. Severity was classified using PASC, based on blood pressure, serum potassium, and plasma aldosterone. Kobayashi score was used to estimates the probability of bilateral PA. Outcomes were assessed by Primary Aldosteronism Surgical Outcome (PASO) and Primary Aldosteronism Medical Outcome (PAMO) criteria.

**Results:**

Among 120 patients (56% female), 52 underwent unilateral adrenalectomy and 68 received medical therapy. Most diagnoses (84%) occurred in the past decade. Median ages were 32 years at hypertension onset and 53 years at PA diagnosis. Most patients presented hypokalemia (75%), more frequent in lateralized than bilateral PA (85% vs 66%; *P* = .03). Overall, 98% of patients had moderate or severe PASC, more common in lateralized than bilateral PA (61.5% vs 36.8%; *P* = .01). Severity correlated with aldosterone levels, hypokalemia, and antihypertensive medications (all *P* < .0001), but not with diagnostic delay. Higher Kobayashi score was associated with bilateral PA. Adrenalectomy resulted in high rates of biochemical success, while medically treated patients showed predominantly partial clinical improvement.

**Conclusion:**

Brazilian patients with PA presented substantial diagnostic delay and predominantly moderate to severe disease. PASC and the Kobayashi score may complement subtype assessment when adrenal venous sampling is unavailable.

Primary aldosteronism (PA) is a common cause of secondary hypertension, with a clinical spectrum ranging from mild to severe, in the latter, profound hypokalemia may occasionally require urgent intervention (1, 2). Once thought to be rare, PA is now recognized to account for approximately 10% of all hypertensive cases and up to 20% among patients with resistant hypertension (3). Despite its prevalence and the availability of effective targeted therapies, PA remains markedly underdiagnosed in clinical practice (2, 4).

Once PA is confirmed, the key management decision is whether patients should undergo adrenalectomy or receive medical treatment with mineralocorticoid receptor antagonists (MRA), depending on the presence of lateralized or bilateral aldosterone hypersecretion (5, 6). Although computed tomography (CT) is widely used in clinical protocols for investigating adrenal lesions and may support decision-making in many cases involving a single, larger nodule, its sensitivity remains limited for detecting small adenomas, bilateral adrenal hyperplasia, and characterizing incidental adrenal nodules (7). Consequently, adrenal venous sampling (AVS) remains the gold standard for subtype diagnosis. However, AVS is invasive, costly, requires specialized interpretation, and is not widely available (8-10).

Primary Aldosteronism Severity Classification (PASC), which utilizes 3 simple indicators, such as serum potassium, blood pressure severity and number of antihypertensive drugs, and degree of plasma aldosterone concentration (PAC), was recently described and provides a semi-quantitative assessment of PA severity based on these widely applicable clinical and biochemical parameters (11). As a clear correlation was found between the PASC and the probability of lateralized PA, this classification may help primary care physicians prioritize AVS when evaluating patients for surgery or treatment with MRAs. By providing graded guidance for AVS, the PASC could support more individualized and efficient clinical decision-making. Another validated clinical prediction model designed to estimate PA subtyping is the Kobayashi score, a simple clinical tool that incorporates variables such as serum potassium, PAC, aldosterone to renin ratio (ARR), female gender, and adrenal imaging findings; lower Kobayashi scores are associated with greater likelihood of lateralized disease and surgical cure after adrenalectomy (12).

Standardized criteria have been used to assess clinical and biochemical outcomes in patients with PA after adrenalectomy or medical therapy with MRAs, classifying treatment response as complete, partial, or absent success. The Primary Aldosteronism Surgical Outcome (PASO) evaluates outcomes after adrenalectomy in patients with lateralized PA based on blood pressure control, medication use, potassium levels, and hormonal normalization whereas the Primary Aldosteronism Medical Outcome (PAMO) assesses treatment response according to improvements in blood pressure, unsuppressed renin and potassium normalization during medical therapy (13, 14).

Two previous Brazilian studies described prolonged diagnostic delay and high prevalence of hypokalemia at the diagnosis (15, 16). One of these studies addressed postoperative renin and aldosterone secretion recovery after adrenalectomy in patients with unilateral PA (15). The other evaluated the association between *KCNJ5* somatic mutations and hypertension remission after adrenalectomy (16). Since both studies had been conducted before the development of the PASC, the distribution of disease severity across lateralized and bilateral PA in Brazilian patients using this clinical tool has not been evaluated. Furthermore, although the Kobayashi score has been proposed as a clinical tool to estimate the probability of bilateral disease before AVS, its relationship with PASC has not been evaluated in PA patients. Therefore, the present study aimed to characterize disease severity using the recently proposed PASC classification, to investigate its relationship with PA subtype and the Kobayashi score, and to evaluate treatment outcomes using standardized PASO and PAMO criteria.

## Patients and methods

Our cohort consisted of 120 patients with PA followed at the University Hospital of Ribeirao Preto Medical School at the University of Sao Paulo, Brazil from January 2000 to December of 2025. The Ethics Committee of the Institution approved the study, and all participants signed the informed consent form (protocol no. 80891224.8.0000.5440).

Clinical and laboratory data were retrospectively collected from the medical records of patients. We adopted the protocol for PA clinical diagnosis and etiology differentiation according to Endocrine Society's clinical practice guidelines (5, 6). Patient blood samples were collected for baseline PAC and direct renin concentration (DRC) assays between 09:00 to 10:00 hours, after 2 hours in the upright position followed by 10 minutes seated. Patients had normal sodium intake diet and hypokalemia, if present, was corrected. Drugs affecting PAC and DRC were withdrawn for at least 4 weeks. Aldosterone suppression tests were not performed in patients with suppressed DRC, PAC of ≥20 ng/dL, and spontaneous hypokalemia (15).

PAC and plasma DRC were determined by chemiluminescence immunoassays using DiaSorin kits (Liaison, Stillwater, MN, USA). The limit of detection for the aldosterone immunoassay at a 20% coefficient of variation (CV) was 2.2 ng/dL. The limit of detection of DRC was 2.0 mIU/L. The intra- and inter-assay CVs were 4.8% and 6.7% for aldosterone and 5.8% and 12.8% for DRC (15).

After screening, all patients performed fine-slice CT protocol for the adrenal glands and, among them, 46 (38.3%) patients also underwent AVS protocol with continuous cosyntropin infusion. The AVS protocol consisted in measuring cortisol concentrations after ACTH stimulation from the adrenal and peripheral veins to confirm successful catheterization, using as selectivity index [SI] ≥ 5. To characterize lateralization, we used the cortisol-corrected aldosterone ratio from high-side to low-side (lateralization index [LI]) ≥ 4 (5, 6). In accordance with the Endocrine Society Clinical Practice Guidelines, patients younger than 40 years with spontaneous hypokalemia, suppressed renin, PAC ≥20 ng/dL, and a unilateral adrenal lesion ≥1 cm without contralateral abnormalities were considered to have lateralized PA and underwent adrenalectomy without AVS. Patients with no lesions on adrenal imaging, presenting chronic kidney disease, severe comorbidities, or other contraindications were considered as probably bilateral PA and managed medically even without AVS (5, 6).

The PASC was applied to our cohort, with each component categorized into 3 severity grades (I-III), corresponding to 1 to 3 points (11). For serum potassium: Grade I (K ≥ 3.5 mEq/L), Grade II (3.0-3.5 mEq/L), and Grade III (K < 3.0 mEq/L). Basal PAC: Grade I (6-9.9 ng/dL), Grade II (10-19.9 ng/dL), and Grade III (≥20 ng/dL). For treated patients, blood pressure was classified as: Grade I included those controlled with 1 to 2 antihypertensive drugs; Grade II included those controlled with 3 drugs; and Grade III included patients requiring more than 3 drugs or those uncontrolled despite ≥1 medication. Each factor contributed 1 to 3 points, and the total score was calculated by summing the points across the 3 domains. PA severity was classified into mild (3 and 4 points), moderate (5-7 points), and severe (8 and 9 points) (11).

We also applied the Kobayashi score to our cohort, a clinical prediction tool developed to estimate the probability of bilateral PA before AVS. The score incorporates 5 routinely available clinical variables: serum potassium above 3.9 mmol/L (4 points) or between 3.5 and 3.9 mmol/L (3 points); the absence of a unilateral adrenal lesion on CT (3 points); baseline PAC <210 pg/mL (2 points); ARR <620 pg/mL/ng/mL per hour (2 points); and female sex (1 point), yielding a total score ranging from 0 to 12. Higher scores indicate a greater likelihood of bilateral disease, whereas lower scores are associated with unilateral PA. Patients with a score of 8 to 12 have approximately a 93.5% probability of bilateral disease, while a score of 0-1 indicate a 15.3% probability of bilateral disease (12).

The multicenter PASO criteria were used to evaluate clinical and biochemical outcomes after adrenalectomy for lateralized PA (13). Clinical success is based on postoperative blood pressure and antihypertensive medication, whereas biochemical success is based on correction of hypokalemia and normalization of the aldosterone to renin ratio (ARR). Complete clinical success corresponds to normotension without antihypertensive medications, partial success when blood pressure improved and/or medication burden was reduced; and absent success when blood pressure remained unchanged or worsened. Complete biochemical success indicates normalization of potassium and ARR; partial success when partial biochemical improvement was observed; and absent success when biochemical evidence of PA persisted.

For bilateral PA, we applied the PAMO criteria to assess clinical and biochemical outcomes after treatment with MRAs (14). Similar to PASO, PAMO classifies clinical and biochemical responses as complete, partial, or absent. Clinical outcomes were classified as complete success when blood pressure was controlled; partial success when blood pressure or medication requirements improved; and absent success when hypertension remained uncontrolled. Biochemical outcomes were classified as complete success when potassium levels and renin response normalized during therapy; partial success when biochemical correction was incomplete; and absent success when biochemical abnormalities persisted despite treatment.

Statistical analysis was performed using GraphPad Prism 10 software (version 10.4.1). Continuous data were expressed as mean, median, and range values. Continuous numerical variables were analyzed using the Mann-Whitney U test. The chi-square test (χ^2^) was used for the analysis of categorical variables. A significance level of *P* = .05 was considered.

## Results

Table 1 presents the clinical and biochemical data of PA patients. A total of 120 patients (56% female) were included; 52 underwent surgery due to lateralized PA and 68 received clinical treatment due to bilateral PA or because surgery was not feasible owing to clinical contraindications or patient refusal. Among the 52 lateralized PA patients, 15 underwent AVS resulting in 10 confirmed lateralized PA, 1 with bilateral PA, and 4 with inconclusive results. The remaining 37 fulfilled guideline criteria for adrenalectomy without AVS. Of the 68 medically treated patients, 37 underwent AVS, with successful catheterization in 22 cases (20 bilateral PA and 2 lateralized PA, who declined surgery), while 15 were inconclusive; the remaining 31 were treated medically without AVS.

**Table 1:** Clinical and biochemical data at primary aldosteronism diagnosis

Characteristics	Total (n = 120)	Lateralized PA (n = 52)	Bilateral PA (n = 68)	*P*
Gender (F)	67 (56%)	20 (38.5%)	23 (33.8%)	.9
Age at hypertension diagnosis (y)	32 [26-39]	35 [26-40]	31 [25-39]	.6
Age at PA diagnosis (y)	53 [41-61]	49 [38-59]	54.5 [47-62]	.**03**
Delay in PA diagnosis (y)	16 [9-25]	14 [7-21]	22 [11-25]	.**008**
BMI (kg/m^2^)	29.7 [26.2-33.7]	29.3 [24.8-33.0]	31.0 [27.0-34.6]	.09
Systolic BP (mmHg)	150 [133-170]	150 [130-170]	152 [139-173]	.7
Diastolic BP (mmHg)	90 [80-100]	90 [80-110]	90 [80-100]	.3
DDD pretreatment	5.0 [3.4-7.2]	5.0 [3.3-7.1]	5.3 [3.5-7.3]	.8
Aldosterone (ng/dL)	26.3 [16.7-48.1]	45.4 [23.7-78]	19.2 [15.2-28.9]	**<**.**0001**
Renin (mU/L)	2.0 [2.0-3.6]	2.0 [2.0-3.2]	2.0 [2.0-3.7]	.1
Aldosterone/Renin (ng/dL/mU/L)	9.6 [6.3-23.8]	19.6 [8.2-34.3]	7.1 [4.2-11.1]	**<**.**0001**
Lowest potassium (mmol/L)	3.0 [2.6-3.5]	2.6 [2.3-3.1]	3.0 [2.8-3.6]	.**0002**
Aldosterone/Potassium (ng/dL/mmol/L)	7.7 [5.6-19.7]	19.6 [7.7-34.0]	6.0 [4.4-8.6]	**<**.**0001**
Creatinine (mg/dL)	0.9 [0.8-1.2]	0.9 [0.8-1.2]	0.9 [0.8-1.2]	.6

Bold values indicate statistically significant results (*P* < .05).

Abbreviations: BMI, body mass index; BP, blood pressure; DDD, defined daily dose; F, female; P, comparison between patients with lateralized and bilateral primary aldosteronism; PA, primary aldosteronism; y, years.

**Figure 1:**
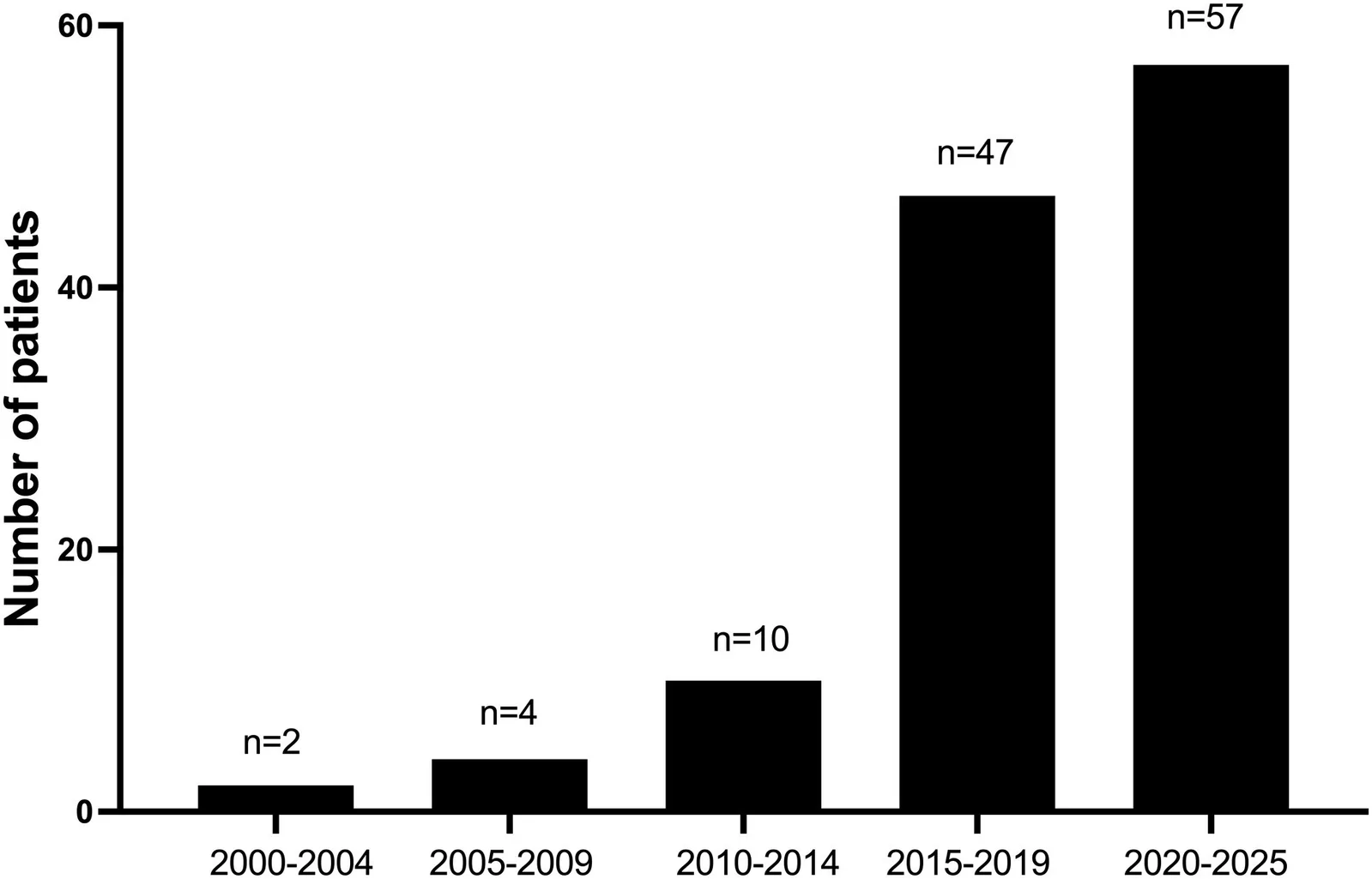
Number of patients diagnosed with primary aldosteronism in a single tertiary center in Brazil over the past 25 years.

Hypertension began at a median age of 32 years [26-39], whereas the diagnosis of PA occurred at 53 years [41-61]. Patients with bilateral PA were older at diagnosis (53 years [45-58] vs 48 [37-59], *P* < .03) and had a delayed PA diagnosis compared to lateralized PA (22 years [11-25] vs 14 [7-21], *P* = .008). Hypokalemia was observed in 75% of patients, being present in 85% of patients with lateralized PA and in 66% of those with bilateral disease (*P* = .03). Most diagnoses (84%, n = 101) were performed in the past 10 years (Fig. 1). Comparing the prevalence of hypokalemia and the diagnostic delay of PA in patients diagnosed between 2000 and 2015 with those diagnosed between 2016 and 2025, we observed no significant differences (90% vs 72%, *P* = .1; 13 vs 17 years, *P* = .2, respectively).

**Figure 2:**
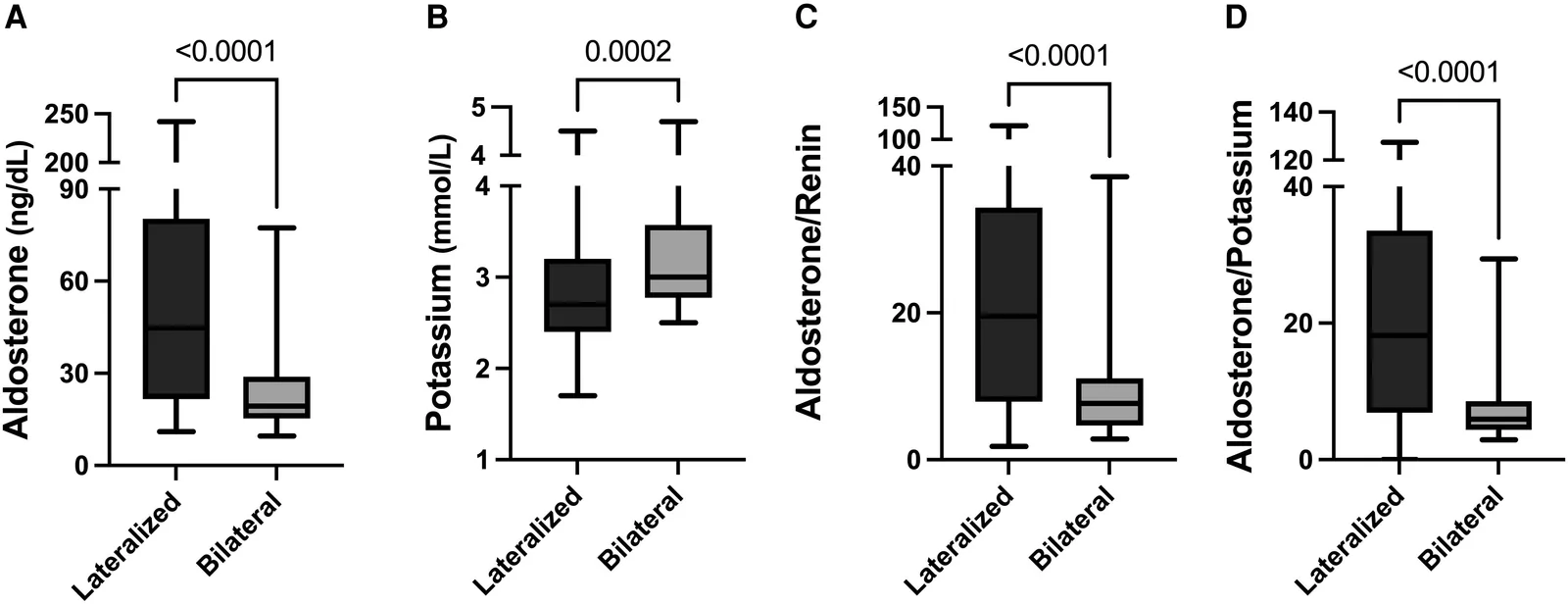
Plasma aldosterone levels (A), serum potassium (B), aldosterone to renin ratio (C), and aldosterone to potassium ratio (D) in the patients with lateralized and bilateral primary aldosteronism.

Patients with lateralized PA showed higher PAC (45.4 ng/dL [23.7-78] vs 19.2 [15.2-28.9], *P* < .0001), lower potassium (2.6 mmol/L [2.3-3.1] vs 3.0 [2.8-3.6], *P* < .0001), higher ARR ratios (19.6 [8.2-34.3] vs 7.1 [4.2-11.1], *P* < .0001) and higher aldosterone/potassium levels (19.6 [7.7-34.0] vs 6.0 [4.4-8.6], *P* < .0001) compared to bilateral PA (Fig. 2). At diagnosis, the bilateral PA group had a higher prevalence of type 2 diabetes (55% vs 25%, *P* = .03) and dyslipidemia (71% vs 44%, *P* = .03).

After adrenalectomy was performed in lateralized PA patients (n = 52), one-third had complete clinical control with antihypertensive medication withdrawal. The others experienced significant reductions in the number of antihypertensive medications compared to patients with bilateral disease (1.0 [0.0-3.0] vs 3.0 [3.0-5.0], respectively, *P* < .0001) and in daily antihypertensive dosage (2.0 [0.0-3.4] vs 4.7 [3.7-6.9], respectively, *P* < .0001). Consequently, surgically treated patients had better systolic blood pressure control than medically treated patients (130 [120-140] vs 140 [120-160] mmHg, *P* = .04). The median spironolactone and amlodipine doses were 50 mg/day and 7.5 mg/day, respectively, in lateralized PA, and 75 mg/day and 8.5 mg/day, respectively, in bilateral PA.

**Figure 3:**
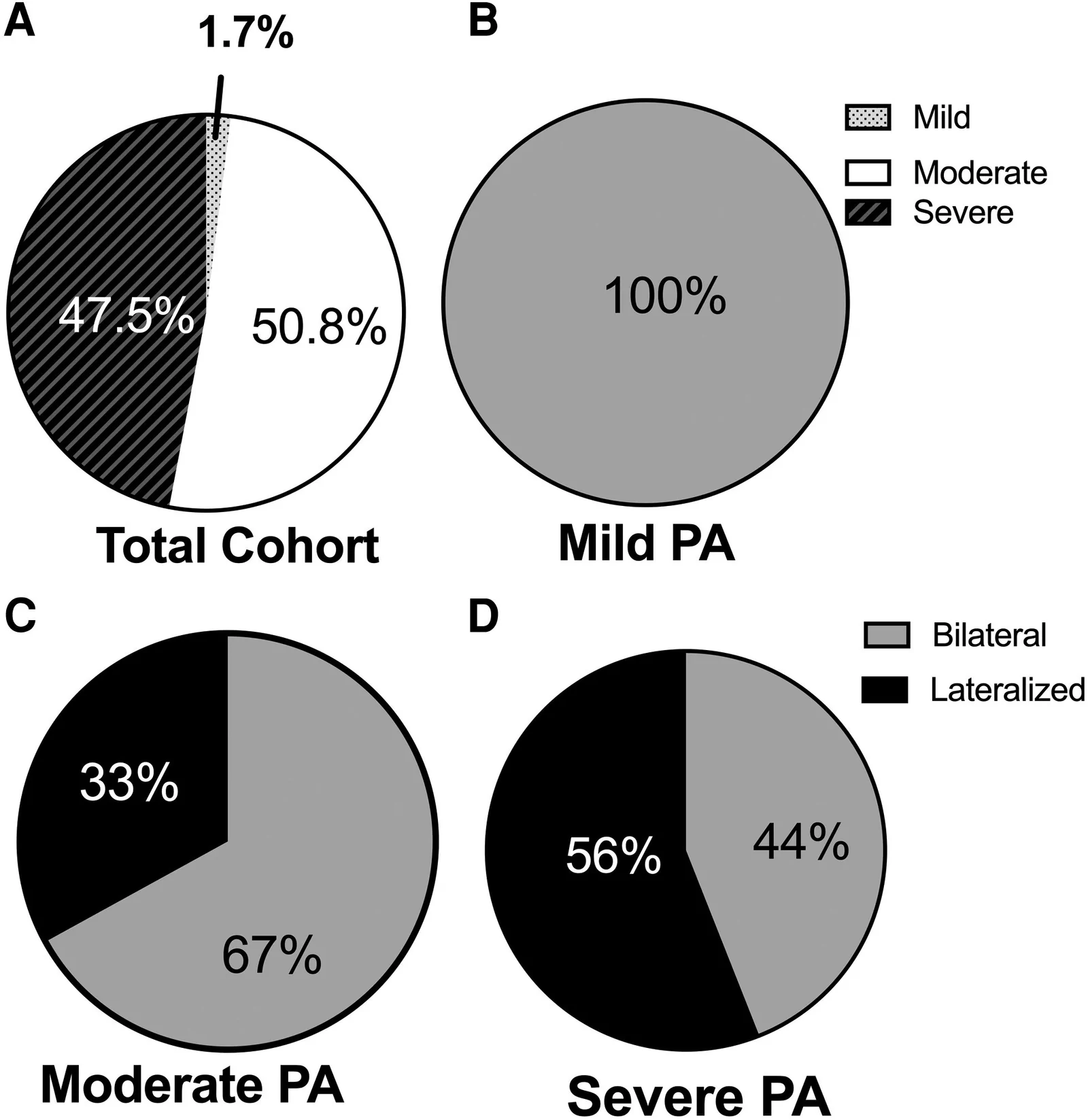
Distribution of lateralized and bilateral subtypes of primary aldosteronism (PA) according to the PASC severity classification in the Brazilian cohort. (A) Overall distribution of mild, moderate, and severe PASC forms in the total cohort. (B-D) Distribution of PA subtypes within each PASC severity category: mild (B), moderate (C), and severe (D). In the overall cohort, 50.8% of patients had moderate PASC, 47.5% had severe PASC, and 1.7% had mild PASC. Among patients with mild PASC, 100% presented bilateral PA. In moderate PASC, 67% had bilateral PA and 33% lateralized PA, whereas in severe PASC, 44% had bilateral PA and 56% lateralized PA.

Overall, the PA severity classification was 1.7% for mild (n = 2), 50.8% for moderate (n = 61), and 47.5% for severe (n = 57) (Fig. 3A). Combined moderate and severe cases accounted for 98.3% (n = 118) of the total. Among patients with mild PASC, 100% (2/2) presented bilateral PA (Fig. 3B). In moderate PASC, 67.2% (41/61) had bilateral PA and 32.8% (20/61) lateralized PA (Fig. 3C), whereas in severe PASC, 43.8% (25/57) had bilateral PA and 56.1% (32/57) lateralized PA (Fig. 3D).

Severe disease was more frequent among patients with lateralized disease than in those with bilateral disease (32/52 [61.5%] vs 25/68 [36.8%]; Fisher's exact test, OR = 2.75, *P* = .01). To minimize potential misclassification, we performed an additional analysis restricted to patients with successful AVS (10 lateralized and 20 bilateral PA). The findings were consistent with the main analysis: 80% of lateralized PA patients had severe PASC and 20% moderate PASC, whereas bilateral PA patients were predominantly classified as moderate (65%), followed by severe (30%) and mild (5%).

PA severity was positively correlated with aldosterone concentration and the number of antihypertensive medications required, and it was inversely correlated with potassium levels (*P* < .0001). Patients with mild PA had a median aldosterone level of 14 ng/dL, potassium level of 3.8 mmol/L, and required a median of 3 antihypertensive medications. In moderate PA, the median aldosterone was 19 ng/dL, potassium level 3.2 mmol/L, and the median number of antihypertensive medications 3, while patients with severe PA presented with markedly higher aldosterone median levels (43.0 ng/dL) and lower potassium levels (2.7 mmol/L) and required a median of 4 antihypertensive medications.

Between 2000 and 2015, only moderate (47%) and severe (53%) PA cases were identified, whereas mild cases emerged after 2016 (2%), with moderate (51%), and severe (47%) (*P* = .4). The median PA severity score was slightly higher in the earlier period (8 [7-9] vs 7 [6-8]), although disease severity was not associated with diagnostic delay.

**Figure 4:**
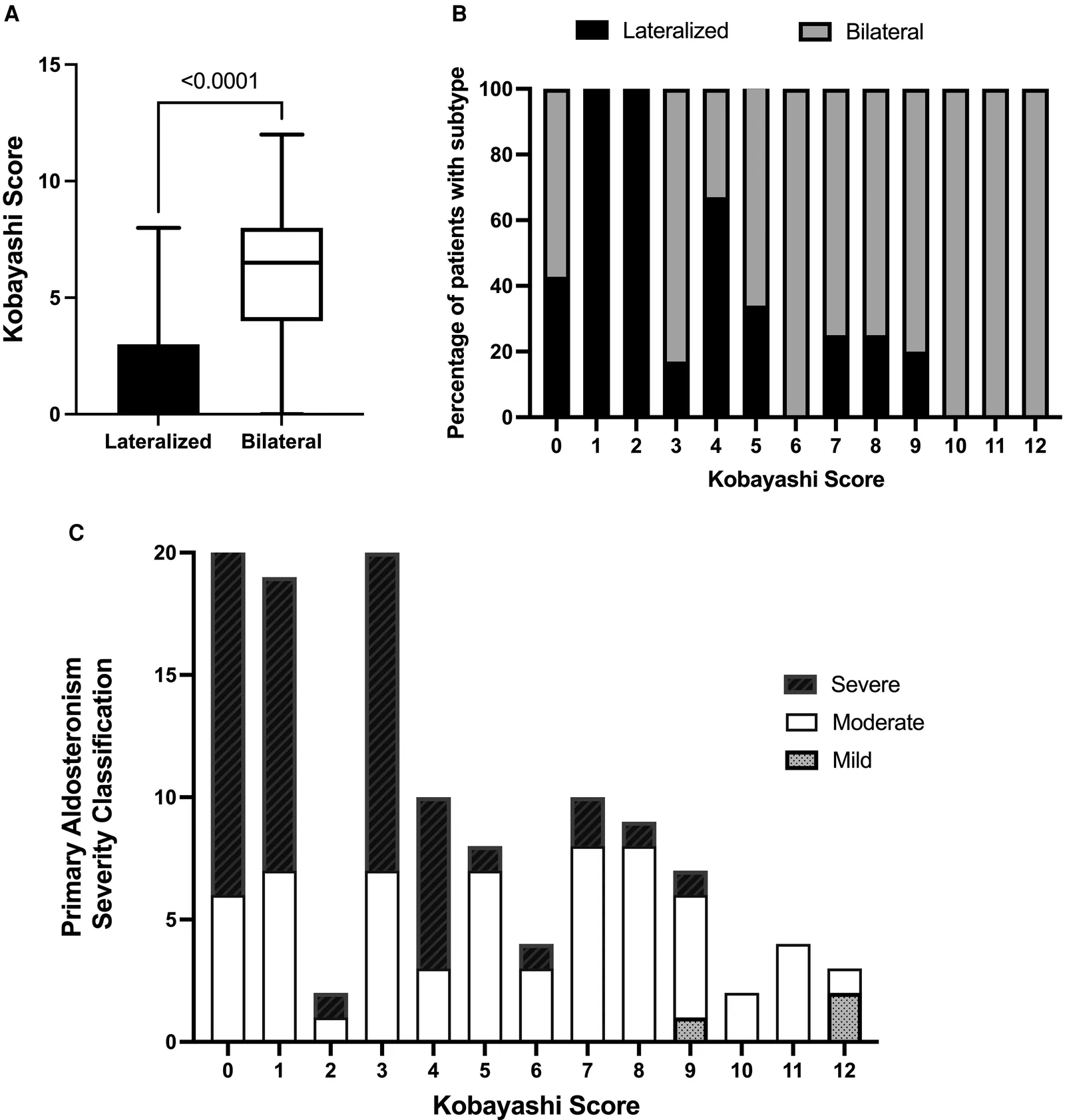
(A) Kobayashi score distribution in patients with lateralized and bilateral primary aldosteronism subtypes. (B) Percentage of patients with lateralized and bilateral primary aldosteronism subtypes in each Kobayashi score. (C) Number of patients in each severe, moderate, and mild forms of Primary Aldosteronism Severity Classification (PASC) in the Brazilian overall cohort according to the Kobayashi score.

The Kobayashi score was higher in the bilateral PA group (6.0 [3.0-8.0]) compared to the lateralized PA group (1.0 [0.75-3.0], *P* < .0001) (Fig. 4A). Of the lateralized PA patients, 92% presented a Kobayashi score <5 whereas 61% of those with bilateral PA patients presented a Kobayashi score ≥5 (*P* < .0001) (Fig. 4B). Of interest, among the 20 patients with bilateral PA confirmed with successful AVS, 7 patients had a Kobayashi score <5. Overall, the clinical, biochemical, and imaging findings of these 7 patients were indeed more consistent with bilateral than lateralized PA. Clinically, the median age at diagnosis was 55 years, similar to that of the bilateral PA group (54.5 vs 40 years, *P* = .03). Biochemically, median aldosterone (31.8 ng/dL) and potassium (2.9 mmol/L) concentrations were intermediate but closer to those observed in bilateral PA than in lateralized PA (Table 1). Imaging was also not suggestive of unilateral disease, with 5 out of 7 patients showing either normal adrenal glands or bilateral adrenal thickening, and 1 patient presented with a 1.4-cm left adrenal nodule and contralateral thickening. The remaining patient had one isolated nodule and the AVS was appropriately indicated.

An inverse significant association was observed between PA severity and the Kobayashi score (Fig. 4C). Kobayashi scores decreased progressively with median of 12, 5, and 1 while PASC severity increased from mild, moderate, and severe PASC, respectively (*P* < .0001). Patients with a Kobayashi score <5 predominantly presented severe PA (71%), whereas those with a score ≥5 more frequently had moderate PA (77%; *P* < .0001). This association remained significant in both lateralized and bilateral PA. Among lateralized PA cases, severe disease predominated in patients with a Kobayashi score <5 (69%), while moderate PA was more common in those with a score ≥5 (86%; *P* = .006). Similarly, in bilateral PA, severe disease was more frequent in patients with a Kobayashi score <5 (77%), whereas moderate PA predominated among those with a score ≥5 (75%; *P* = .002).

PASO score was obtained in 47 and 45 out of 52 patients for clinical and biochemical criteria of control, respectively, in patients after adrenalectomy for lateralized PA. Among these, 18 (38%) patients had complete clinical success, 28 (60%) had partial, and 1 (2%) had absent clinical success criteria. Regarding biochemical criteria, 35 (78%) patients had complete, 9 (20%) had partial, and 1 (2%) had absent biochemical control. There was no significant association between PA severity and outcome.

PAMO score was obtained in 48 and 41 out of the 68 patients for clinical and biochemical criteria success, respectively, in patients after medical treatment for bilateral PA. Among these, 1 (2%) patient had complete clinical success, 36 (75%) had partial, and 11 (23%) had absent clinical control criteria. Regarding biochemical criteria, 30 (73%) patients had complete, 3 (7%) had partial, and 8 (20%) had absent biochemical control.

## Discussion

The present study provides a comprehensive characterization of severity of PA patients with lateralized and bilateral disease in a Brazilian cohort. Over a 25-year period, nearly all diagnosed cases at this tertiary referral center presented moderate to severe phenotypes. The predominance of severe clinical and biochemical features, together with the long interval between hypertension onset and PA diagnosis, highlights the ongoing challenges in timely diagnosis and recognition of milder forms of the disease in clinical practice.

One interesting aspect to point out is that there was an increment of PA diagnoses made in the past decade, likely reflecting improvements in screening practices. Another major finding of the present work is the prolonged interval between hypertension onset and the diagnosis of PA, with a median delay of approximately 2 decades. The prolonged interval between hypertension onset and PA diagnosis is multifactorial. Factors such as serum potassium levels before diagnosis, previous antihypertensive treatment, the timing of aldosterone and renin measurements, and a possible initial normotensive phase may have contributed to the prolonged diagnostic delay. These data were not available in our study because patients had been managed for many years in primary care before referral to our tertiary center, precluding a retrospective assessment of these potential contributors, which likely also reflects the recognized underdiagnosis of PA in routine clinical practice. Delayed recognition remains a consistent report in the literature, despite increasing awareness of PA as the leading cause of secondary hypertension (3, 17, 18). Indeed, an international survey reported that 35.6% of patients experienced a diagnostic delay of 5 years or more, particularly women and individuals with higher prevalence of comorbidities (18). These data strongly suggest recommendation in screening strategies. Notably, screening rates in guideline-recommended populations remain below 2% in the United States, contributing to persistent underdiagnosis and delayed recognition of PA (19).

Similarly, we observe a decrease in the prevalence of hypokalemia in the last decade (90% vs 72%). In the entire cohort, hypokalemia was found in 75% of patients, particularly among those with lateralized disease. Although historically considered a hallmark of PA, hypokalemia has been reported in contemporary cohorts in 20% to 40% of cases (3, 20). The high hypokalemia incidence observed in our study is in agreement with previous published Brazilian studies, suggesting patients with severe hyperaldosteronism, probably due to the long delay of hypertension onset and the PA diagnosis (15, 16). These findings highlight the need to increase awareness among healthcare professionals, mainly family physicians, general physicians and cardiologists, for diagnosis and timely intervention to minimize morbidity and mortality associated with PA (21, 22).

Patients with lateralized PA exhibited a more severe biochemical phenotype than those with bilateral disease, characterized by greater aldosterone excess, more pronounced hypokalemia, and stronger renin suppression. These differences are consistent with the more robust aldosterone secretion described in lateralized aldosterone-producing adenomas (23). On the other hand, metabolic comorbidities were more frequent in the bilateral group, supporting evidence that chronic aldosterone excess contributes to insulin resistance, endothelial dysfunction, and increased cardiometabolic risk (24). Our findings align with reports suggesting that prolonged exposure to milder but sustained hyperaldosteronism may play an important role in cardiometabolic derangements.

The Kobayashi score, a clinical prediction tool used to help estimate the likelihood of lateralized vs bilateral disease before AVS, showed that patients with bilateral PA exhibited significantly higher while surgically treated patients demonstrated lower Kobayashi scores. Our data on the inverse association between lower Kobayashi scores and more severe PA reinforce its value as a marker of clinically significant lateralized disease and underscore the importance of accurate preoperative evaluation to optimize long-term outcomes (12, 25). Taken together, our findings on bilateral PA confirmed with successful AVS in patients with Kobayashi score <5 reinforce that AVS remains the gold standard for PA subtype classification. Furthermore, the Kobayashi score was not developed to replace AVS but rather to estimate the probability of unilateral or bilateral PA and assist in subtype prediction.

PASC, a recent classification system used to stratify PA according to disease severity, showed in our study a distribution that substantially differed from the international multicenter validation study (11). Analyzing a total of 2593 cases with PA from 26 centers around the world, PASC classification reported 13.9% mild, 63.0% moderate, and 23.1% severe PA. Our Brazilian cohort reported a lower prevalence of mild PA (1.7%) and a remarkable enrichment of severe disease (47.5%). Similarly, in the PASC cohort study, services such as Tampere, Saint-Petersburg, and Nijmegen reported no mild cases. In contrast, Japanese services, such as Yokohama1, Yokohama2, and Sapporo, reported that nearly half of their cases were classified as mild (51.3%, 45.5%, and 44.8%, respectively) (11). These divergences likely reflect a combination of referral center bias and differences in healthcare resources and screening practices, resulting in delayed recognition of PA and more advanced disease at diagnosis in some underdeveloped countries. Altogether, our and the Japanese studies showed that severe PA was strongly associated with lateralized disease whereas bilateral PA predominantly exhibited mild to moderate phenotypes. Increasing disease severity correlated with progressively higher aldosterone levels, lower potassium concentrations, and greater antihypertensive treatment, supporting the clinical relevance of this classification system. Of note, PA severity was not associated with diagnostic delay, suggesting that disease severity might depend on other intrinsic pathophysiological factors than by disease duration alone.

Our results suggest that severe PASC is associated with lower Kobayashi scores and lateralized disease, whereas moderate PASC is more frequent in patients with higher Kobayashi scores and bilateral disease. The concordant findings in the whole and in the subgroup with successful AVS suggest that potential subtype misclassification might not influence the main results. Of note, subtype misclassification remains possible when AVS is not performed or is inconclusive—and may even occur despite successful AVS. Thus, our data support the ability of PASC and the Kobayashi score to help the discrimination of disease severity and PA subtype definition, respectively. The inverse relationship between PASC and the Kobayashi score reinforces the concept that disease severity and subtype prediction represent complementary dimensions of PA management. From a practical perspective, the combined use of PASC and the Kobayashi score may assist in prioritizing patients for AVS in healthcare systems where access to specialized procedures is limited. Nevertheless, neither tool replace AVS, which remains the gold standard, whenever surgical treatment is being considered. Therefore, these tools should be viewed as complementary rather than competing approaches.

These findings are congruent with the pathophysiological characteristics of aldosterone-producing adenomas, which typically display higher aldosterone output than bilateral hyperplasia (26). The association of lower Kobayashi scores and severe PA is also consistently observed in lateralized PA and reinforces its utility as a clinical predictor of surgically curable disease (12).

In our study, adrenalectomy for lateralized PA resulted in substantial reduction in antihypertensive medication need and improvement of blood pressure control, as previously described (26, 27). The rates of complete clinical and biochemical success, according to PASO criteria, observed in our study (38% and 78%) are similar to those of multicenter analyses, which reported complete clinical success in approximately 37% to 50% and complete biochemical success in up to 94% of patients (13, 28). These findings emphasize that even in the absence of full clinical remission, biochemical control confers important cardiovascular benefits by eliminating aldosterone-mediated end-organ damage. In contrast, patients with bilateral PA treated medically exhibited poorer blood pressure control and lower potassium levels during follow-up. Our results mirror prior evidence demonstrating that MRA therapy is frequently limited by suboptimal dosing, side-effects, and interindividual variability in response (14). Only one patient (2%) achieved complete clinical control based on PAMO criteria, highlighting the challenges inherent to exclusive medical therapy and suggesting that more aggressive titration or adjunctive approaches may be warranted. Indeed, when PASO or PAMO remains with partial or absent clinical control, in our center, if additional blood pressure lowering is needed, we always used optimized MRA and added a long-acting calcium channel blocker, followed by an angiotensin-converting enzyme (ACE) inhibitor or angiotensin II receptor blocker (ARB).

Our study has some limitations. The retrospective design and the tertiary referral center setting may have introduced selection and information biases, potentially favored the inclusion of more severe PA cases and limited the generalizability of the findings. Temporal heterogeneity, especially in long study periods during which diagnostic criteria, assays, imaging methods, and treatment approaches may change. Another limitation is the retrospective nature of PASC on aldosterone and potassium concentration, the number of antihypertensive medications, as previously acknowledged by its developers. Consequently, caution should be considered when interpreting disease severity based on this classification. Nonetheless, the detailed phenotype, application of PASO and PAMO criteria, and long-term follow-up at the same hospital enhance the strength of our observations.

In conclusion, the PASC classification effectively stratified PA according to disease severity and showed a strong association with the Kobayashi score and PA lateralization. Severe PASC was predominantly associated with lower Kobayashi scores and lateralized disease, whereas moderate phenotypes were more common among patients with higher scores and bilateral PA, supporting the complementary clinical value of both classifications in disease characterization and therapeutic decision-making. Collectively, our data highlight the persistent challenges in optimizing the diagnosis and management of primary aldosteronism, underscoring the need for broader screening strategies to enable earlier recognition and targeted treatment, thereby reducing the excess cardiovascular and renal morbidity associated with aldosterone excess and preventing progression to more severe, potentially irreversible complications.

## Data Availability

Data generated during and/or analyzed during the current study are available from the corresponding author on request.

## Abbreviations

ARR: aldosterone to renin ratio
AVS: adrenal venous sampling
CT: computed tomography
DRC: direct renin concentration
MRA: mineralocorticoid receptor antagonist
PA: primary aldosteronism
PAC: plasma aldosterone concentration
PASC: Primary Aldosteronism Severity Classification
PASO: Primary Aldosteronism Surgical Outcome
